# Wounds of the Chest

**Published:** 1918-08

**Authors:** 


### WOUNDS OF THE CHEST

There has not been published a more authoritative treatise on war wounds of the chest than the report of a session of the Research Society which appears in this number of *War*

*Medicine*. All phases of the subject were not dealt with in this symposium, but those considered were discussed by surgeons of high attainments who spoke from a large experience with the French and British Armies in treating wounds of the chest.

Major General Sir John Rose Bradford discussed the diagnosis, dealing particularly with the methods for determining the anatomical conditions present, and for deciding the very important question of the presence or absence of infection in wounds of the chest.

The following subjects were discussed by other speakers: "Wounds of the Chest", by Colonel A. F. Soltau; "Operative Results of Early Surgical Treatment", by Médecin-Major Pierre Duval; "The Treatment and Management of Psychoneuroses", by Lieutenant-Colonel Gordon Holmes; "Operative Treatment in Chest Surgery", by Major A. L. Lockwood; "Removal of Foreign Bodies from the Chest under Fluoroscope", by Dr. Petit de la Villéon; and "Secondary Surgical Treatment of Chest Wounds", by Professor Tuffier.

### When to Operate

The discussion brought out an interesting difference of opinion as to when and where to operate in wounds of the chest. Colonel Soltau contended that many cases would recover without operation, which should not be performed without definite indications; while Major Lockwood and Médicin-Major Duval urged immediate operation. It developed in the discussion that the statistics show about the same results from both methods of treatment.

The question of immediate operation in chest wounds would seem to depend upon a number of considerations, involving the skill and experience of the surgeon, the facilities for operating, and the condition of the patient. The surgeons who have had large experience, and have therefore acquired great skill in operating upon lung wounds, will probably get the best results by the immediate operation, if the patient's condition warrants it; while surgeons of limited experience, and uncertain technic, will save more patients by waiting until there are definite indications for an operation. A consideration in time of battle, when a great many wounded have to be treated, is whether or not a surgeon shall devote an hour or more to a man who is very seriously wounded, with the chances perhaps against recovery, if in doing so he must delay treatment of a number of other men who have slight wounds which would not become infected if they were looked after immediately, but which may develop gas gangrene, or other infection, if allowed to go a few hours without the first dressing. From the military viewpoint it is very important for the slightly wounded man to get back to the line as soon as possible, but for that reason alone the seriously wounded man must not be neglected.

These are questions for each individual surgeon to decide; but he can get facts that will help him in dealing with wounds of the chest, if he will read the reports of the Research Society Meeting.

